# Illustrations of the Theory and Practice of Ventilation, with Remarks on Warming, Exclusive Lighting, and the Communication of Sound

**Published:** 1845-01

**Authors:** 


### BRITISH AND FOREIGN

### MEDICAL REVIEW,

FOR JANUARY, 1845.

### PART FIRST.

### Analytical and Critical Reviews.

### Art. I.

*Illustrations of the Theory and Practice of Ventilation, with Remarks on Warming, Exclusive Lighting, and the Communication of Sound.* By D. B. Reid, M.D.—*London*, 1844. 8vo, pp. 452.

In this climate and state of society, which subject so large a portion of the population to an in-door life, the subject of ventilation has claims the importance of which it would be difficult to overrate, as they press on all classes of the community. This "age of great cities" congregates human beings into huge masses; and whether they are heaped up in manufactories, or in ships of war, or in churches, chapels, halls, or theatres, or in close cabins, rooms, tenements, or streets, they are all subjected to one great cause of disease—each man poisoning the atmosphere both for himself and his neighbour.

Since the discovery of gases, and of the law that all gases tend to diffuse themselves through each other, ventilation has become a branch of chemistry, and promises to be one of those by which this great science may be applied to the highest purposes of physical discoveries—the improvement of the health and consequently of the well-being of the community. It is fortunate that the necessity which our legislators felt for the comfort of their own persons in the House of Commons, led them to seek the assistance of Dr. Boswell Reid, whose strong manly intellect and untiring energy and zeal are well fitted to apply to practical purposes great scientific principles. The work before us is the result of his investigations. We have previously alluded to its publication: we return to it, in order to lay before our readers those parts which are of special interest to us as medical practitioners. For although the practical management of ventilation must be left to architects and builders, yet, in our investigations as to the causes of the diseases we are called upon to remedy, we should be fully aware of the evils which impure air produces, the probable sources of those evils, and the possibility of removing them.

*Pure and impure air.* No one can doubt the truth of Dr. Reid's observation, that there is great indifference as to the quality of the air we breathe. Its influence is not actually realized, owing to its producing no marked or tangible effect on our external senses; otherwise, those who have every luxury at command, and who spare no pains to gratify the demands of the grosser appetite for food and drink, would not be comparatively thoughtless as to that "finer, more ethereal, and purer food, which has access directly, and without any intervening digestive process, to the living blood."

"It is not generally understood that in innumerable public and private assemblies, churches, theatres, schools, &c., an atmosphere is often breathed for hours continuously which is as foul and offensive, compared with the air that is congenial to the lungs, as the water of the Thames at London Bridge contrasted with a pure mountain spring. It is no exaggerated statement to affirm, that the greatest scourge with which this and so many other climates is affected, viz. consumption, owes its origin more to ignorance of the laws of health connected with the peculiarities of exposure to alterations of air and temperature, and to the severity of local draughts, than to any disadvantages connected with the local state of the atmosphere which cannot be met with proper care and attention; that numerous other diseases, particularly catarrhs, rheumatisms, and pleurisies, often spring from this cause: that a depreciated tone of mental vigour, as well as of bodily health, may, in endless examples, be traced to the same cause; that the most deadly plagues that have ever appeared have been aggravated, if not caused, by want of cleanliness and ventilation, and that the ordinary typhoid fever of this country almost invariably originates under similar circumstances: that hospitals imperfectly ventilated have in some cases proved a curse instead of a blessing to the miserable patients who have been conveyed to them; that public establishments are known to the medical profession, where, at one time, from the same cause, no case of compound fracture ever recovered, few or none survived the amputation of a limb; mortification attacked every wound, however trivial, while the prostration of strength became so great, that men who had at first stood the severest operations without a murmur, subsequently cried like children from the slightest pain; and that cases have actually presented themselves where the apparently lifeless corpse, subdued and oppressed far more by the atmosphere with which it was surrounded, than to the disease to which it was supposed to have fallen a victim, has actually been known to revive after removal to the dead-room, a separate apartment, where the play of a wholesome atmosphere, flowing unrestrictedly upon it, revived the fading flame of life after it was to all appearance gone, and where health and strength were ultimately restored; that the practice in hospitals has been accompanied with the most decided reduction of mortality as the ventilation has been improved: that even in cities, generally, the mortality has regularly diminished as the external ventilation of the streets has been placed on a better and more systematic footing, by increased attention to cleanliness, and by affording free channels for the passage of air." (pp. 9-10.)

The houses of the rich suffer most from the gaseous products of their kitchens, sculleries, drains, and smoky chimnies, drawn into their halls and staircases, and thence into their rooms; and also from excessive illumination at night, which, when the rooms are ill-ventilated, produces headach and languor. Where gas-lights are used the atmosphere produces soporific effects in many in a very short time. This is the case in innumerable shops and counting-houses. The ventilation of the houses of those whose station places them above the wants of the common necessaries of life is very defective, and Dr. Reid imagines that a few elementary lessons on ventilation would in these cases be very valuable. The miserable condition of the houses of the very poor need not be dwelt upon by those who have our opportunities, as medical men, of becoming personally acquainted with these deficiencies. Dr. Reid observes, that where food and clothing are deficient, ventilation need not be expected; for heat is more essential than fresh air; and defective ventilation, by reducing oxygenation, "preserves warmth, stupefies the feelings, and allays the pangs of hunger."

The deficient means of ventilation in our churches, especially in those with galleries, is a fruitful cause of ill health. Dr. Reid has visited numerous churches in hot weather, to observe the effects of bad air on the congregations, and the following graphic sketch is evidently drawn from the life:

"In those churches in which I have watched the progress of the influence of vitiated air, as the service proceeded, on individuals whose constitutions were not previously rendered dead to the influence of pure air by the state of the atmosphere to which they were accustomed at home, a slight and marked flush in the countenance usually appeared in a short time; this was soon succeeded by a sense of heat and oppression, and a tendency to sleep, more or less marked, according to the condition of the atmosphere, and the extent to which the attention was engaged. The soporific influence in some cases was a source of annoyance to the conclusion of the service, but was succeeded in others by a reaction. The *vis medicatrix naturæ* did not remain inactive. The pulse rose, the circulation was accelerated, the brain became stimulated, and relief was at the same time afforded by the insensible perspiration (which had been arrested by the state of the air) becoming gross and sensible. Attention could now be sustained, but headach more or less severe was the usual consequence, and liability to dangerous colds and rheumatisms, where the body was suddenly exposed to the chilling influence of the external atmosphere, while still affected in the manner described." (p. 42.)

In a moral point of view the ventilation of churches is important. The great difficulty or complete inability which so many feel to keep up the necessary attention to the services, and for which inattention one set of hearers (those whose consciences are most susceptible,) accuse themselves and another set attribute to the dullness of the clergyman, or to the sameness of the routine, depends very much on a cause over which they have no control. The brain is so poisoned by the inhalation of air impregnated by carbonic acid gas, that it is unequal to its functions; so that the exercise of mere attention, not to speak of thought, becomes difficult, and to many almost impossible. And thus the direct object of church attendance is frustrated in a twofold manner: for, as Dr. Reid observes, the same noxious agent which produces the careless indifference of the congregation subdues the preacher's own energies. As so many churches are now building, and the medical man in the country is, or ought to be, an authority on a point in which the health (physical and mental) of so many are concerned, it should be remembered "that the mere packing of the greatest possible number within the smallest possible space, ought not to be the only rule adopted, even in the humblest churches. Health and power of attention to devotional exercises form primary considerations." (p. 45.)

"In the general disposition and arrangements of new churches, the removal of pews, the closing of all side windows, illumination by the roof, the exclusion of galleries, and the adaptation of the spire as an instrument of ventilation, have often forced themselves upon my attention as objects of great practical importance, which might be well worthy of consideration, and often introduced with such modifications as peculiar localities may require." (p. 45.)

The effect on the living of burial in towns is a subject on which the medical practitioner is called to pronounce an opinion, and Dr. Reid is strongly opposed to the practice :

" In ordinary circumstances, the principal part of the body is slowly resolved into gases, and these tend ultimately to form carbonic acid, nitric acid, and water; the most offensive and dangerous products are those that are formed before the full oxygenation ensues." (p. 50.)

" In the great majority of cases, in a climate such as this country presents, the whole area of a long-used and crowded churchyard may be justly considered, in a practical point of view, as a manufactory of pestilential exhalations, perpetually in operation, and so abundantly supplied with the materials of production, that at no time, probably, is their discharge entirely arrested, however marked it may be at some periods compared with others, according to the state of the weather. And, since the laws of the diffusion of gases have been more thoroughly understood, and the practice in the majority of churchyards made known, no one who considers the facts connected with this subject can hesitate to believe that even where the graves are considerably deeper than usual, this newly appreciated power of diffusion must operate incessantly with extreme effect in communicating deleterious products to the superincumbent atmosphere, so long as there is anything to be decomposed." (p. 51.)

" In some churchyards, I have noticed the ground to be absolutely saturated with carbonic acid gas, so that whenever a deep grave was dug, it was filled in a few hours afterwards with such an amount of this gas, that the workmen could not descend without danger." (p. 52.)

The arrangements for purifying burial-grounds and cemeteries must much depend on their special localities ; but Dr. Reid gives some hints which are generally appreciable :

" The mere removal of the large gravestones that cover so many churchyards, the introduction of proper footpaths, and the cultivation of flowers and shrubs, luxuriant in their vegetation, but not lofty or prone to prove any serious impediment to the free flow of air or the action of light, would be of incalculable value in numerous situations, absorbing the products of decomposition, and replacing in general by their purifying influence a better atmosphere than could otherwise be sustained." (p. 52.)

It should be borne in mind that noxious vapours discharged from manufactories may be injurious to the health and comfort of the neighbourhood, and yet the atmosphere within the building may be wholesome; so that the good health of those employed on the premises is no proof that the air without has not been injured. The noxious principles of such emanations may be destroyed, or altered by fire, or may be retained by the action of water, or of water charged with lime; but the difficulty is to render these processes economical, for in many cases such processes would more than consume the profits of the manufac turers. Diagrams are given explaining some of Dr. Reid's plans.

Smoke is one of those increasing nuisances to which the attention of Parliament has been directed. On looking at the murky cloud which overhangs by day our great cities, and contrasting it with the clear canopy above them at sunrise, or with the habitual atmosphere of continental cities where wood alone is burned, and taking into consideration the effect of the constant inhalation of such impure air on the lungs themselves, in which the carbon accumulates until they become blackened, and the inevitably filthy state of the skin which coal-smoke produces in all those who cannot afford, or whose habits are against complete cleanliness, it is impossible not to wish and to look forward to the time when science may show the way by which this carbon may be turned to useful purposes, and its present waste may appear as prodigal as the ancient plan of emptying the scrapings of the streets into the nearest river. But besides the injury which smoke must produce on the health, there is the discomfort attaching to existence in so filthy a medium, soiling our houses, and furniture, and books, and works of art, and marring the artistic effect of our public buildings and monuments.

Dr. Reid thinks that the manufacturers who produce the largest quantities would find it highly economical and advantageous to consume it, that is, when they use larger boilers. "But when too small boilers are employed in proportion to the power required, the necessary amount of steam is not always to be obtained, except by excessive firing, when a large quantity of smoke is almost invariably produced."

"No cases demonstrate in a more convincing manner the extreme importance of the general introduction of elementary science as a branch of education, than those connected with the use and disposition of fuel, where a little more knowledge on the part of those who manage the fires would be attended with the most beneficial results, and enable them to extend indefinitely improvements in the use of fuel, and in the construction of furnaces." (pp. 63-4.)

It would be a great point gained (says Dr. Reid) if a familiar test for estimating the purity of the air could be applied with the same facility as the thermometer for ascertaining the temperature. He has employed various means, but he has found none so convenient for ordinary purposes, when individuals unacquainted with chemical analysis were to apply them, as lime-water, or a solution of baryta. Carbonic acid is the principal impurity produced by respiration and combustion, and where it predominates other impurities may be expected. A little lime-water or baryta-water in an ounce phial immediately indicates the presence or absence of carbonic acid by the precipitation or non-precipitation of a compound of the earth and the carbonic acid which the air of the apartment may contain. The quantity will depend on the rapidity with which the precipitate takes place. The worst air Dr. Reid has met with was in a printing-office lighted by gas, where the lime-water became coated with a crust as soon as poured out. As there is carbonic acid in common air a white deposit will form on long exposure to the purest air. Experience is necessary, and a practised eye will judge of the purity of the air by merely agitating lime- or baryta-water in a wine glass. In preserving specimens the best mode is to seal the phial hermetically by fusing its neck. For more precise experiments, Dr. Reid has invented a carbonometer, by which the current of impure air is passed through the alkaline solution, and compared with the results obtained by the passage of the same quantity of common air through the same medium. We must refer to Dr. Reid's book for the explanation of the instrument, which is rendered easily comprehensible by diagrams. In all such experiments the operator must take care that the breath from his own nostrils does not vitiate the results.

Ventilation requires an ingress of air from the external atmosphere as well as an egress. It is by the new air entering that the old air is expelled. But in many buildings, where attention has apparently been paid to ventilation, an egress alone exists, and in others large apertures in the ceilings lead to air-tight roofs, where the vitiated air, if the weather is cold, is cooled, and again descends, instead of being conveyed in a distinct channel into the open air. The perfection of ventilation consists in a free supply of air so completely in harmony with the body that its operation is not perceived. Currents or draughts indicate imperfections. It is a common error to suppose, as carbonic acid is a heavy gas, that air vitiated by its evolution in respiration and combustion is also heavy. Carbonic acid gas is only heavy in a concentrated form; but when once mingled with a considerable body of atmospheric air, it does not again separate in consequence of its specific gravity; in proof of which it is found in the air of the highest regions which man has visited. Expired air has, from its heat and moisture, a tendency to ascend; so has air vitiated by combustion: so that, as a general rule, an upward motion should be given to air in ventilation; the air which enters should never return to the zone of respiration. In ordinary apartments a mixed current, lateral at first, and ascending afterwards, is the best. In some instances a forced descending movement is necessary. In his numerous experiments Dr. Reid was satisfied that an amount of diffusion, far beyond what had been previously effected, was necessary; and that, as a general principle, diffusion could not be too universal.

"In a room for an invalid subject to cold or afflicted with consumption, the walls and floor might, if necessary, be rendered entirely porous for the admission of air, and the ceiling arranged in a similar manner for its discharge.

"In ordinary apartments, a great amount of diffusion may be secured by taking advantage of the skirting for this purpose, and in all churches, lecture-rooms, schools, theatres, &c. considerable opportunities are generally presented, particularly in the rising steps, where air can be led in with diffusion.

"In large apartments, occupied by a few individuals only, if the air be admitted at a distance from them, sufficient diffusion may be secured by the extent to which the air may be broken in its course before it reaches them.

"Again, the colder any air used for ventilation, the more important is it to attend to the amount of diffusion.

"An extreme variety of textures have been employed for promoting diffusion, according to the circumstances under which they have been required. Hair-cloth is extremely useful from its perfect elasticity, and from its not being absorbent nor retentive of moisture. Wire-gauze, perforated zinc, and numerous woollen, cotton, hemp, and silk textures have been employed where different materials were required; they may be dyed or painted in imitation of oak, or according to any other pattern, so as to be in unison with the apartment in which they are placed." (pp. 90-1.)

With regard to the power to be employed in ventilation, Dr. Reid concludes that, in a larger proportion of cases, advantage is to be taken of the natural property of vitiated air to ascend, and that when additional force is necessary to sustain the current, no power is so convenient as artificial heat in increasing this natural tendency to ascend. Mechanical instruments, screws, fanners, &c. should be resorted to only when circumstances render these other means too expensive or complicated.

He illustrates this natural ventilation very copiously both by explanations and diagrams. We can merely give an outline of his illustration.

Suppose a room filled with persons standing on a porous floor, with the roof taken off, an upward movement is the consequence; fresh air is introduced below, and the vitiated air passes off above. But an open roof is inadmissible. Protection is required, and the opening above is contracted, and in proportion to the amount of contraction, the temperature of the room, and the numbers on a given space, it becomes necessary to increase the velocity of the discharge. If a shaft or chimney be extended from an opening in or near the ceiling, the volume of warm air that fills it increases the draught; and if this is not sufficient, by kindling a lamp or a fire, or by merely increasing the temperature of the room, additional velocity is given to the column of hot air within the shaft. A chimney, therefore, from its extreme simplicity and from the comparatively trifling attendance it requires, is preferable to machinery. Further, when properly finished at the top, the wind acts as a power, and, without any fire, often determines the ascent of air.

The mode in which the chimney or shaft operates is thus clearly explained.

"Whenever a heated object is placed, as a candle, the air in contact with it immediately expands, bulk for bulk; accordingly, air near it, but not yet expanded, is pressed under the expanded portion, and a current is thus established towards the source of heat, which is sustained so long as any inequality of temperature continues.

"In the same manner, the air around the human frame is always warmed and expanded as it comes in contact with the body, and, in this condition, an atmosphere at a mean temperature, and not agitated by any wind, is usually elevated about five degrees in its temperature, as it rises and escapes above the head.

"If the candle be introduced into a tube, the current of air which is thus established is more rapid in its ascent, unless the tube be extremely wide. The warm air, not being so accessible to the cold, external air, a greater difference of temperature is established, which is accompanied by a corresponding difference of specific gravity and of velocity in the currents." (pp. 94-5.)

If a candle is placed in the centre of a room, a stream of warm air ascends from it, and colder air presses towards it from every side; the upward stream of warm air having reached the ceiling, passes to the circumference, cools, falls, and on reaching the floor, passes again to the central heat. If a mass of ice is substituted for the candle, the air descends from the cold ice, and all the currents are reversed. In the same way a window in cold weather is always the source of descending currents of cold air, independently of any influx of cold air from without. Such descending currents are a common source of discomfort and of disease, particularly of colds, rheumatism, and inflammations. They should be studiously avoided in churches and halls, and public buildings where there are large windows.

"I have seen corridors where nearly a thousand square feet of glass were at a temperature below 32°, thickly covered with hoar frost condensed on the interior surface, and pouring down a continuous stream of ice-cold air, while the general temperature of the rest of the corridor varied from 60° to 70°.

"It would probably be a source of permanent economy were all public buildings with large windows, and in daily use in winter, provided in this manner; much less fuel would then be requisite to warm them in cold weather." (pp. 117-8.)

We must refer to the work itself for the chapter on "the elementary illustrations of ventilation," in which are explained, by clear and numerous diagrams, the action of various schemes, and pass to the remarks on gases, air, and respiration.

Extreme mobility, compressibility, and elasticity (says Dr. Reid), are the leading mechanical properties of the air and other gases. No balance is so nicely adjusted as the pressure of one portion of air upon another. Practically speaking, the air is scarcely ever absolutely quiescent, as the most trifling change of its specific gravity by heat or pressure is accompanied by motion and an endless variety of currents and eddies. In those districts where the alternations of hill and dale, land and water, produce unequal temperatures, the most frequent changes are observed. But besides these causes, vegetation, animal life, volcanic agencies, and electricity act in the same way, and prevent any death-like calm. The important discovery of the diffusion of gases has thrown much light on their properties. No one would have expected (says Dr. Reid) that a light and a heavy gas would mix together in opposition to their natural gravities, where no chemical action ensued between them. It is now proved that all gases tend to diffuse themselves through each other, one gas passing into another as if drawn into a vacuum. Light gases even descend and heavy gases ascend to mingle with each other, though the medium of communication is only through a capillary tube, and a considerable force is exerted as they mix. Bring two different gases into communication, and the result is a homogeneous mixture of the two gases without any change of temperature. The velocity of this diffusion depends on the density of the gases. The lighter one gas is, the more rapidly does the heavier gas diffuse itself through it. Atmospheric air, for instance, diffuses itself into hydrogen more quickly than into carbonic acid. In consequence of this, no gas, however heavy, remains at the surface of the earth, and as Graham has observed, if it were not for this provision, the carbonic acid in the lungs would pass and repass indefinitely in the more minute vessels without being conveyed to the external atmosphere. But by this diffusive principle it darts from the air-cells into the bronchial tubes, from which it is expelled by expiration. On the same principle gases pass through numerous porous solids, and (it is considered) the whole body may be permeated by them. Hence the importance of porous clothing to allow the escape of gaseous exhalations from the surface of the body. It has been proved that some porous solids absorb gases and condense them. Many metals in fine powder absorb large quantities of oxygen ; charcoal can absorb about ninety times its bulk of ammonia ; and soils absorb ammonia, and other substances from the air, which contribute to the growth of vegetables. Thus gases on the earth's surface are subject to two antagonistic powers. By diffusion they are prevented from accumulation in large masses, and by condensation they are reduced to a more useful form where any quantity of their elementary principles is needed. In this condensed form they still retain their gaseous condition, and are supposed neither to be reduced to a solid nor to a liquid state, and the term gaseous condensation is employed.

Moisture is an essential component of atmospheric air. The feeling of damp does not depend on the quantity of moisture alone, but on the tendency of the air to deposit it, and this depends on its temperature. In warm summer weather in this country, there is more moisture in the air than in winter, but it does not feel so damp, as the tendency to deposition is less than in winter. The more moisture there is in the air the larger the amount of air required for the evaporation of the insensible perspiration, and the greater the difficulty for the lungs and skin to cast off the products which are always discharged from them. The 'treacly atmosphere' of warm climates is warm air loaded with moisture, and deposits moisture on the body rather than relieves it by evaporation. Its injury to health is thus evident. In cold climates, the reverse happens; large quantities of moisture are carried away by condensation, and with them a proportion of carbonic acid and other impurities, leaving the residual air more pure. In expeditions to the Polar regions this has been observed.

"When the walls of a crowded apartment run down with moisture, this indicates that the air in contact with them is saturated with moisture, and that they are colder than the air. If they were not, there would be no deposition.

"Again, when a warm south-west wind succeeds a long and severe frost, the same effect is observed. The water that appears does not exude from the walls; it is deposited from the warm air, when its power of solution is diminished by coming in contact with the cold wall." (p. 156.)

The following memoranda explain facts with which we are practically acquainted, although their causes are not always reflected on. The air is almost always colder than the body. The feeling of warmth is owing to the air exerting a less cooling influence than we have been habitually, though unconsciously, used to. The importance of good habits, in accustoming our bodies to cool and fresh air is obvious. The morning air is the freshest and purest from the deposition of moisture during the night, which carries with it many impurities with which it has been contaminated during the day, when it is continually receiving moisture and volatile impurities from the earth. Air on the ocean is saturated with moisture, and contains a little saline matter. Air on the dry land is often deprived of part of its moisture by the absorptive powers of the soil. In spring and summer it contains more oxygen from active vegetation; in autumn, more impurities from putrefaction. It is more impure on imperfectly drained land than on well drained. Clayey soils are less wholesome than gravel, because the latter is more porous, and absorbs impurities more quickly. Sunshine purifies the air by the heat favouring ascending and descending currents, and in promoting oxidation and evaporation. Shade tends to produce stagnation, the air remaining cold on the surface of the earth. The warmth of the air in towns in winter is favorable to health, but this is often counterbalanced by the noxious products of cities.

"In the country, a house situated at a moderate elevation, equally free from the severity of the mountain blast and the stagnation of the valley, not encumbered by trees, nor yet distant from vigorous vegetation on well-drained land, and, above all, so located as to receive the greatest possible amount of the sun's rays in the individual apartments by the arrangement of the windows, a due regard being paid to protection from cold, may be considered as placed in the most favorable circumstances for the promotion of health." (p. 166.)

Winds acquire the qualities of the place over which they pass. Hence the difference to the feelings between the north and the east wind, as contrasted with the south or the west wind.

"The former are essentially cold and dry in general, when compared with the latter; and accordingly, by the free addition of steam, and a proper elevation of temperature, an easterly wind, as it enters any house, may be made to approximate in character to the south-west wind." (p. 167.)

In ventilation, the quantity of fresh air necessary for each individual is the first thing to be determined. In some respects this is a question of expense. The nearest approach of a room to the purity and freshness of the atmosphere would be the most conducive to health, but this air must be warmed so that its purity is sacrificed to warmth, in order to avoid expense. The standard of pure air is unfortunately low, for the air to which an individual is habitually subject becomes his standard, not what is most conducive to his health and strength, and the power of the system in accommodating itself to circumstances is very great.

"The amount of opium, wine, tobacco, ardent spirits, and other substances, which some individuals can habituate themselves to, though only at the expense of impairing their constitutions, is no less remarkable than the varieties of atmosphere, loaded with impurities, in which others can breathe, and even work for years continuously. Perhaps the most singular instance of this kind, exclusive of those met with in polar regions, and in a rude state of society, are to be seen in mines, where it is frequently common for the men to work in an atmosphere too impure to permit a common candle to burn, though an oil lamp, in consequence of its greater tenacity of combustion, may be maintained in action without difficulty. But numerous individuals faint in an atmosphere far less impure than that of some coal-mines, and even death has been considered to have ensued in some instances from vitiated air, though it was not sufficiently impure to extinguish a candle, no impurity but carbonic acid being known to have been present." (p. 179.)

"But confining our attention to an average temperature, and to any ordinary apartment not overcrowded with many persons, and not subject to any peculiar or noxious emanation, it is considered that ten cubic feet per minute should be provided for each individual.

"This estimate is given with much diffidence, and only as an approximation. It is the result, however, of an extreme variety of experiments made on hundreds of different constitutions, supplied one by one with given amounts of air, and also in numerous assemblies and meetings, where there were means for estimating the quantity of air with which they were provided." (pp. 175-6.)

For the modes in which these experiments were made, we must refer to the diagrams and explanations of Dr. Reid's volume.

"If we look to the fact that less than half a cubic foot of air passes through the lungs of an adult in a minute, this estimate may at first appear excessive; but if we remember that, at each expiration, a quantity of air is emitted which mingles with an additional portion of air exceeding largely its own bulk, and that there are twenty such respirations per minute, while provision is also required for the air that affects the surface of the body, and for the endless variety of minor effects produced by furniture, lighting, heating, refreshments, &c., where no peculiar adaptations for these purposes have been introduced beyond those usually observed, it will be seen that the estimate is by no means immoderate. The real question is, not what the constitution can bear, but that amount which is conveniently accessible in ordinary habitations, and which is essential for the wants of the system." (pp. 178-9.)

The connexion between the appetite and respiration, has been amply descanted upon by Liebig, who has shown more fully than his predecessors how much more food is taken and required, when the lungs are supplied with pure and highly oxygenated air. The converse is true, that a low diet diminishes the necessity for much air, and that where there is little air, there cannot be a great appetite for food. Dr. Reid illustrates this by an anecdote. Some years ago, fifty members of one of the Royal Society Clubs at Edinburgh, dined in an apartment he had constructed so that all the products of the combustion of the gas with which it was lighted were removed, and large quantities of a mild air were constantly supplied, and passed in quick succession through the room during the whole evening, variously perfumed with the odours of flowers. Mr. Barry of the British hotel, was familiar with the constitutions of these intellectual sybarites, and amazed the committee the next day by proving to them that the sage who had usually taken two glasses, had consumed his half bottle, and the philosopher whose usual quantum was half a bottle, had taken a bottle and a half; for three times the quantity of wine had been drunk than was usually imbibed by the same party, in a room lighted with gas and unventilated. And what is as curious, both from its scientific interest, and from its illustrating one of the customs of modern Athens, Dr. Reid, with his usual statistical gravity and earnestness, assures his readers that "minute inquiries afterwards assured me that no headache had followed" this dinner, but adds "nor were any of the members aware that they had partaken more heartily than usual;" which obliviousness admits of two interpretations.

Another example of the same fact is even more striking. Some manufacturers have stated that their men "struck for wages where a good system of ventilation had been introduced, as their former wages were insufficient to procure them the increased amount of food which their appetites now demanded," and indeed "no argument is more common against ventilation among many, than the expensive appetite it maintains."

The cheap refreshment rooms in London are ill-ventilated. "Many a hard-worked clerk too often imagines he has had enough for his support, because he has taken all that his appetite permitted; whereas the saturated atmosphere in which he dines may have reduced his appetite by a half, and made him contented with an inadequate supply." We need not expose the importance of this connexion between the air breathed and the appetite in a medical point of view. It explains the tonic effect of pure air, and throws some light on the reason why our higher classes who live so much more than the middle ones in the open air, and when within, are in much larger rooms, can live so much higher with more impunity.

The amount of moisture in the air affects the supply required. Where the air is moist and warm, a much larger quantity is required to carry off the pulmonary and cutaneous exhalations than when the air is dry, so that 100 cubic feet of moist and warm air do not give so much relief as a few cubic feet of dry.

Dr. Reid has found that a temperature of 65° is most generally agreeable in rooms not over crowded, the atmosphere moving in a gentle stream. Its effect on the bodily feelings is greatly influenced by the rate at which it moves. The air, as has been before stated, is almost always below the heat of the body, but if it is stagnant it does not produce much cooling effect as a minute quantity comes into contact with the body in a given time. But if the wind blow freely, many successive quantities approach the body in the same time, and a sensation of cold is produced. " An air at a very low temperature is not dangerous in a calm, but if it begins to blow, the face and limbs may soon be frost-bitten." Thus in ventilation, if the air was below the temperature of the body, the same atmosphere which is offensively cold, might be rendered pleasant by reducing its velocity, and a sultry atmosphere might be rendered cool by increasing its velocity. If the air is hotter than the body, increasing its velocity, increases the sensation of heat on the same principle.

The moisture which the air contains is next in importance to its temperature. The body and limbs form a vast surface, covered with millions of pores, from which moisture is continually escaping, or is absorbed, according to the state of the air. Hence the relief or the oppression of the system shows the state of the air. The drier the air the greater its power of dissolving this moisture. In winter, the colder the air the freer from moisture, as it deposits what it held in solution. Hence its effect in making the skin harsh and dry. The mucous linings of the nostrils and fauces feel dry and dusty, a disposition to cough ensues, as they are deprived of part of the moisture essential to their functions. Hence the necessity of communicating moisture to air which is artificially warmed in winter. Where there are great numbers in a room, it is difficult to maintain a pleasant temperature, unless by a great movement of air. Dr. Reid has found that air introduced at the floor at 60° became 70° and 80° before it reached the head, if the crowd was great.

" In the lower deck of a man-of-war, I have repeatedly noticed a difference of 11 and 12 degrees in winter between the temperature at the floor of the lower deck, where the air was permitted to enter, and that of the atmosphere about five feet above on the same deck, where the hammocks were suspended. In railway coaches, ordinary mail coaches, and other vehicles, during winter, a difference to the same amount may be frequently observed, if the windows be shut and no ventilation permitted, between the temperature of the air around the head, and that in contact with the foot. And, in general, it may be noticed, that the play of aerial currents often presents very great differences of temperature within spaces exceedingly near, and even within the same apartment, so that, in the erect posture, the feet may be exceeding cold, while the head is oppressed with heat; or, air may be tolerable in the body of a theatre, and oppressive, in the extreme, in the galleries; or it may be only comfortable in the latter, and intolerable from its cold below." (pp. 185-6.)

The demand for heat varies in different individuals, according to constitutional peculiarities, diet, clothing, excitement, exercise, time of life, and manner in which they are occupied. It should be lower and lower the more recently any food has been taken, and the larger the quantity. When exhausted by long-continued watching and attention to objects involving no bodily exertion, the heat may be increased many degrees with advantage. In spring and summer the warmth enables the air to gather moisture from the earth and sea, and to hold it in more perfect solution. The magnitude of the function of perspiration and the influence of moisture in retarding, and of dry air in quenching, its operations, explain the risks which are run in change of climate without that change of diet and habits which is in unison with it.

Dr. Reid glances only on the effects of light and electricity on the body. The marked influence which we know that light produces on plants in promoting their powers of absorbing carbon and throwing out oxygen, render it not impossible that light may be a more important agent in our own economy than we are aware of. But precise observation is wanting. Dr. Reid mentions that in a barrack in St. Petersburg, three cases of disease occurred on the shady side of the building to one on the other, though the rooms on both sides communicated, and the discipline, diet, and treatment were the same. In experiments on his own respiration, he found that he recovered more rapidly when exposed freely to light, as well as to air; and he is convinced, from experiments in white, black, green, and other painted rooms, that "a very different influence is produced upon the body by rooms differently painted." We quote "these guesses at truth" to stimulate others who are calculated for such inquiries to investigate this important subject more fully. The London Electrical Society are collecting facts on the connexion between the electrical state of the atmosphere and the presence of diseases,—a connexion with which invalids with susceptible nervous systems, who are attentive self-observers, are very conscious of; no electrometer indicating with more certainty the presence of electricity than their feelings in thundery weather. On this obscure subject we may expect that chemistry will eventually throw much light. The analogy between nervous power and electricity, the facility with which some persons part with their electricity, as their hair often indicates, and the power of retaining it which others possess, so that one man, when placed on an isolated stool, and charged with an electrical machine, gives out again the electricity so freely that a conductor gives no indications of its presence, whilst another becomes fully charged; the effects of certain clothing, and of metallic rings, in rheumatic neuralgias, are isolated facts; but the discovery of the general laws on which they depend must not only improve the hygienic treatment of many diseases, but be essential to the rational application of electricity or galvanism as a therapeutic agent. For at present it is an empirical remedy; and, like all such, it occasionally cures, but in the majority of cases fails; and as we know not why it cures or why it fails, we have no rules to guide us in its application. Can we be surprised at any want of success when it may be indiscriminately thrown into the body of one who has too much already, or of one who has too little, and perhaps without any attention to the course of the current, so that an operator may be counteracting nature by driving his electricity through the nerves in a wrong direction?

*Vitiated air.* In a practical point, the following impurities require the most attention?

1. Carbonic acid. Air containing even one per cent. of this gas is unwholesome, but by habit a much larger quantity may be borne, just like excess of wine or beer, without any immediate ill consequences, but ultimately with diminution of appetite and strength. Dr. Reid has been exposed to air of a high temperature containing ten per cent. of carbonic acid, but he does not think he could have resisted its deleterious action unless it had been gradually deteriorated. The weaker the constitution the less easily is this borne. The symptoms produced by even a small quantity are headache, and various degrees of oppression. On recovering from insensibility produced by this gas, the individual is unable to moan or to move for hours, although sensible, and his head feels like a mass of lead chained to a rock.

2. Carbonic oxide. This is produced by imperfect combustion, where there is much carbon. Coal-gas is offensive to many constitutions, even in small quantities. Fire-damp contains less carbon than coal-gas prepared artificially, and has less smell, though both are equally obnoxious to life; miners, however, work in an atmosphere where there is a considerable proportion of fire-damp.

3. Sulphuretted hydrogen is the common product of the putrefaction of animal matter, and of sea-water, subjected, in a limited atmosphere, to the action of organic matter. Lime or mortar in drains evolve it when in contact with some acid. Even one 15,000th part has been known to be injurious.

4. Sulphurous acid. This gas, the same as is produced by burning sulphur, escapes in many chemical operations from manufactories.

5. Hydrochloric (or muriatic) acid is disengaged in many processes. This escapes daily from the chimneys of British barilla, or soda manufactories, the effect of which is apparent on the surrounding vegetation. Dr. Reid has tested it in the air two miles from the lofty chimney from which it was discharged. It is readily absorbed by water, and in this way sometimes got rid of. In small streams it may destroy fish, but where there is a great flow, and the water impure, it may be beneficial.

6. Ammonia is a common impurity where there is animal matter undergoing decomposition.

7. Arsenic, copper, and lead are often suspended in the air, as the state of vegetation proves, in the neighbourhood of manufactories, where they are used. Tallow candles hardened by arsenic so as to look like wax, must be very injurious. Besides these impurities, there are those of malaria, and the principles of contagious diseases, which have escaped the chemist's power of analysis, and the more obvious mechanical impurities from the degradation of roads, the ordinary impurities left in streets, smoke, &c. Some articles of furniture give an unpleasant smell to rooms, especially when unventilated. In the present state of science, the nose of the practitioner is the only test of these sources of impurity, to which he should be alive when investigating the causes of disease.

*Purification of the air.* If it be possible, offensive emanations should be destroyed at their source, thus striking at the root of the evil. When this is impossible, as in many manufactories, a system of ventilation, and an adaptation of proper chemical agents is necessary, such as require professional knowledge. Dr. Reid gives a few practical observations on fumigation, which are generally useful.

Lime-whiting owes its efficacy to quicklime absorbing carbonic acid from the air, or from organic substances with which it is mingled, and to its causticity which destroys animalculæ, and decomposes animal or vegetable matter. The more fresh and recently slaked the better. When it loses its causticity, it has become carbonated like chalk, and comparatively useless, and fresh whitewashing is necessary. Chlorine acts by dehydrogenating. It decomposes organic compounds by uniting with their hydrogen and forming muriatic acid. Chloride of lime owes its efficacy to the small quantities of chlorine which it evolves for a considerable time, and is the most convenient form for supplying chlorine. The addition of sulphuric or muriatic acid increases the evolution of chlorine, but although a small quantity does not irritate the lungs, large quantities are very irritating, and should never be evolved in an inhabited apartment. Nitrous or nitric acid vapours are convenient, as they may be used without removing the patient. For this purpose, nitre may be thrown into an equal weight, or two thirds of its weight of sulphuric acid in a small cup, resting in sand, and held over the fire in an iron ladle until there is a slight appearance of white fumes. A greater heat volatilizes the sulphuric acid and produces excessively irritating vapours. In uninhabited rooms, such as cellars, the most powerful fumigation may be used by pouring sixty parts of common salt with one hundred of nitre into two hundred of sulphuric acid heated in the same way, or by boiling a mixture of nitric and muriatic acid: chlorine and nitrous acid are evolved. But this is not so desirable as lime-whiting. Vinegar does not purify the air, but rather disguises bad air by its more agreeable smell. Ammonia purifies by uniting with carbonic acid, and on this account is very refreshing to many. Excessive heat decomposes and renders innocuous infectious matters. For this purpose, where other means cannot be used, rooms when uninhabited should be heated by coke or charcoal to 212°.

*Artificial atmospheres.* We do not recollect to have seen the importance of ventilation more forcibly illustrated than by the following simple arithmetical calculation :

"What frequent repetition of any ordinary prescription can ever approximate to,

| | | | |
|---|---|---|---|
| 20 | distinct and separate impulses in | | 1 minute. |
| 1200 | ,, | ,, | 1 hour. |
| 28,800 | ,, | ,, | 24 hours. |

and all these acting, not upon a secondary organ—not subject to any intermixture with the food or products of digestion—but conveyed directly to the blood in the lungs, and presented to an area many times exceeding that of the surface of the body!" (p. 217.)

It also encourages us to hope that chemistry in connexion with practical medicine may teach us to introduce our remedies through this most natural inlet with better success than has yet attended our efforts in this direction. For we all must confess that inhalations as commonly employed are of little real service in the treatment of pulmonary diseases. Smoking tobacco and stramonium are almost the only exceptions. But to breathe through the tube of an inhaler for a short time several times a day, or to use medicated vapour baths once a day, or even less frequently, are shown by this plain preamble to be very insufficient proceedings, hardly worthy of the name of remedies, mere placebos by which the practitioner appears to do something when he really effects nothing. Besides the regulation of the temperature, dryness or moisture of the apartments, the rational plan must be to impregnate the air which is habitually inspired with gaseous remedies, and so to carry off with facility the noxious products which are exhaled from the body. Dr. Reid gives a list of twenty-three gaseous ingredients which may thus be employed ,and which can be introduced, and in determinated quantities, with facility in apartments and air-baths he has constructed, in which the chemicals are conveyed to a ventiduct, through which all the air of the room or bath enters. Such experiments must be tested in our hospitals.

"By constructing a few chambers in every hospital, where the quality of the air that passes the zone of respiration might be entirely under control, and medicated, heated, dried, moistened, cooled, and applied in any quantity, as circumstances might dictate, a more specific power would be obtained, capable of being applied advantageously in numerous cases of disease." (p. 217.)

Part Third of this volume is on the production and communication of heat and light; and amongst much matter which is chiefly of interest to the architect or to those who are planning new residences, there are observations which may be of service to the medical practitioner.

All clothing of close texture, which prevents exhalation from the skin, and renders it damp from condensation, is injurious. The advantage of woollen stuffs, and especially flannel, in this country, where there are great changes both in temperature and moisture, is owing to their warmth, as powerful non-conductors, and their porosity. Air-tight boots are injurious to invalids; and we would add, cloth boots, the tops of which are lined with mackintosh, which keep the feet of the invalid damp and cold from retaining and condensing the perspiration. The following remark is judicious:

"Were the amount and quality of clothing more frequently adapted in this country to the state of the atmosphere to be encountered, whether at home, in the open air, in churches, or in assemblies, innumerable cases of disease would be prevented." (p. 227.)

The point to be aimed at is to avoid sudden transitions of temperature, and draughts, and to keep up an agreeable but not an oppressive warmth.

No one is more aware than those in our profession how much more refreshing sleep is when the clothes are taken off; so that none but very "young hands" refuse a bed and prefer a sofa: Dr. Reid explains the fact by the comparative freedom with which cutaneous exhalation goes on in bed when the day clothes are removed.

While admitting the utility and importance of close stoves of a low temperature, especially of Dr. Arnott's, in particular cases, we heartily join with Dr. Reid in his praise of an open fire for common sitting-rooms. It circulates the air and ventilates the room; it enables you to apply just the quantity of warmth you wish, as you can regulate both its size and briskness and your distance from it; the warmth it gives is more stimulating and exhilarating from its accompanying light, which acts upon the mind by its beauty, cheerfulness, and life, so that it has the effect of a living companion.

What is called the cottage grate is the most agreeable fireplace, and the most scientific. It should be placed low down, with an ashpit immediately beneath (so that the fire is fed by air entering in front only), and with a blower above, which can be easily raised or lowered. The object is to heat the floor as warm air rises; and the brick back readily radiates the heat, and does not, like iron, conduct much of it up the chimney. A plan with diagrams is given.

Dr. Reid greatly praises coke, soft-burnt, in small pieces not bigger than a walnut, wihch is not deprived of all its gas, and kindles easily. It gives a cheerful and agreeable warmth, without smoke. Some object to coke, as if it produced bad air; but it only produces the same gas, minus the smoke of coal, and on this account its escape into the room is not so soon recognized and prevented. The cottage grate is well adapted to coke. The advantage of stoves is that they are cheaper, cleaner, less limited in their action, and require less attendance; but it must always be remembered "that they will not ventilate rooms like an ordinary fire, and the ventilation must be attended to independently." They are more economical, but they are less wholesome, for their economy depends on the small quantity of air which is removed by them. Dr. Ure, in his evidence before the House of Commons, said he is sure that if Dr. Reid had ever experienced the effect of stove-heated rooms of Russia, Sweden, or Germany, he would deprecate their imitation in this country, as the greatest of evils. Dr. Reid replies with the true "nemo me impune lacessit" feeling, "I must tell Dr. Ure that I have examined well-managed stoves abroad, and that none produce a more mild, genial, and equable temperature." He refers to those used by Berzelius and Mitscherlich as perfect as it is possible to procure. But this is no answer to Dr. Ure. It may be true that scientific men, or those to whom expense is no object, will remedy the unhealthy closeness of a stove warmth, by sufficient ventilation; but we can conceive nothing more prejudicial to the health than the stove-heated rooms of the poorer classes in the countries Dr. Ure mentions. Enter the cottage of a German peasant in the depth of winter, and you are almost suffocated with the close hot-house heat, and stench, and you leave it in a state of perspiration. Everything is sacrificed to cheap warmth; and those who do not lead a life of hard labour in the open fields (which, fortunately for them, is the case with the majority of the men, and of nearly all the women,) show its baneful effects in their pallid appearance. In this country, the use of close stoves in counting-houses, and rooms of business where individuals are kept many hours of the day to a close sedentary employment, is often most prejudicial to the health; for the almost inevitable tendency is to neglect due ventilation, especially when the introduction of fresh air diminishes the feeling of warmth. An individual may become accustomed to impure air, so as to be unconscious that it impairs his strength; but the very debility which this produces, renders warmth more essential to his immediate comfort, and leads him to insure warmth, to the neglect of pure air. We can fully agree with Dr. Ure, that the common substitution of stoves for open fires in this country, would be a great evil, whilst agreeing with Dr. Reid that a Berzelius or an Arnott might warm his study or laboratory by the same application of heat, and, without any detriment to his health, he might save much dirt, expense, and attendance. Poets tell us of "skiey influences," and of the silent and unconscious effects of mountains or plains upon the minds of their inhabitants; and it may be asked whether the substitution of a dark black iron mass for the cheery living fire, would not unconsciously make its owner a duller man, and quench his imagination and vivacity. If it is so, however, the inventor of our English stove is a proof against his own invention; though it may be asked by those curious in the action of matter upon mind, whether the gloomy stove-warmed rooms of the Germans have not had some influence on their mind, in driving them into themselves, and leading them to cultivate the world within them, rather than to observe that which is without. But in such an estimate, the pipe should not be forgotten—the most *subjective* in its tendency of all pleasures.

The plan of warming by hot water was invented in France during the last century, but systematized and strongly advocated in this country by the Marquis of Chabannes, twenty-five years ago. The principle on which it acts is that cold water, from being specifically heavier than warm, always descends, and pushes up the warmer water. In this way, a reservoir of water heated at the kitchen fire, connected with similar reservoirs on each story of a house by means of an ascending and descending pipe, warms the whole—a constant circulation going on as long as heat is applied to the boiler below, and lost in the apartments above, which it warms. It is a convenient plan in hospitals for supplying each floor with hot water. Dr. Reid fully illustrates the subject by diagrams. Hot water is more convenient than steam, as any degree of heat below 212° can be obtained; whereas, steam-pipes must either be kept in full action or not in action at all.

Whether light be obtained from oil, candles, or gas, it is gas that is burned; so that a lamp or candle may be regarded as a small gas manufactory, and, like a fire, it deteriorates the air. The amount of light depends on the carbon, in its minute state of division, being heated more or less. If air is supplied too freely, so that the carbon is at once oxygenated, the flame is of a pale blue, as at the base of the luminous cone of the flame of a candle. If the supply be defective, carbon passes off unconsumed as smoke. To obtain the brightest light, the supply of air must be so regulated as to suspend the carbon in the flame for a short time before it is consumed, and thus to heat it to a white heat, like a white-hot cinder in a furnace. This is attempted in the argand and solar lamps, and in the Bude light. The flame of a candle is a hollow cone, within which is unconsumed gas. In the argand lamp, air is introduced into the centre of the flame, so that both its outer and inner surfaces being freely supplied with air, are in an equal state of combustion, and consequently the carbon is more intensely heated.

In the solar lamp, instead of the air being permitted to ascend in the usual manner around the flame, it is made to strike upon that part of the flame where it is most important to have a sufficient supply by means of the curved director or shield. In the Bude light, oil and oxygen were at first used, but now merely air and coal gas, so that it is only an improved gas burner; the gas-burner consisting of concentric rings of tubes perforated in their upper surface, and the gas being heated before it escapes, so that the carbon is more instantaneously heated. Each light, as has been before stated, is in fact a small fire, and in like manner it deteriorates the air. Carbonic acid and water are the common results of combustion; small quantities of uncondensed gas escape; carbon as smoke, and small portions of tallow, oil and other special matters become suspended in the air. Where the amount of light is small, this is of no great importance, but wherever a high degree of illumination is required, these impurities should be carried off by ventilation before they mix with the air of the room. We must again refer to Dr. Reid's diagrams to explain clearly his plans for this purpose. It is our business when consulted to urge their adoption, the arrangements must be left to others.

Part iv contains a copious explanation with diagrams of the processes for ventilation which Dr. Reid has introduced into the present Houses of Parliament.

The House of Commons has been turned into a large pneumatic machine. All the air is introduced into a series of chambers beneath the house through an opening fifty feet square, over which is suspended a fibrous veil to detain any mechanical impurities as soot, 200,000 visible pieces of which it has stopped in one evening. Having passed through this opening, the air is heated, or cooled, or dried, or charged with moisture as may be required, in the various chambers appointed for these purposes, and having attained an equalizing chamber, it passes upwards into the House through the floor, which is perforated with millions of holes, and covered by a hair-cloth carpet, in order to insure its equable diffusion. It escapes above into a foul-air chamber above the roof, and from thence into a channel which conveys it to a shaft, the ventilating power of which is increased by a fire within it. The channel through which it escapes is controlled by a single valve, by which the velocity of the air and its temperature is readily adjusted, and the area of the opening by which the air is discharged, is as large as that where it is admitted. The House is heated to 62° before it is opened, and is maintained at a temperature between 63° and 70°. In extremely warm weather, air at 75° has been rendered agreeable to the feelings, by increasing its velocity. At night during summer after the members have left, cold air—produced by the evaporation of water, by the cold-water apparatus, or even by ice—is drawn through the House. Such is a slight sketch of Dr. Reid's plan. He is so little of an egotist, that he never directly informs us of the success of his plans, though we presume by the continuance of his appointment, that the results are on the whole satisfactory. That he has made the air more wholesome cannot be doubted, but as the individuals subjected to his processes judge more from their own sensations of comfort than from scientific rules, as to the atmosphere best suited to their health and strength, he may not give universal satisfaction. He mentions meeting one member coming out, saying " we shall be suffocated immediately," and another following him with the exclamation " I am shivering with cold;" reminding us of the old story of the travellers on the mountain. And after all a medium atmosphere is the only one he can attempt, and this will strike the honorable member who comes in warm from exercise as oppressive, and another who comes cold from his carriage, as chilly. The speaker and sergeant-at-arms, who remain sometimes ten or twelve hours at a time have their own atmosphere under their own control.

The House is lighted by " exclusive lighting," that is, the gas lights are cut off by glass from communication with the apartment itself. The products of the combustion of each burner are carried off by smaller channels into leading lines of communication on either side of the house, which open into a ventilating shaft. The means best adapted for ventilation have likewise aided in the better communication of sound, a point of the greatest importance. The principal causes of defect were: the great height of the ceiling by which much sound was lost, the soft and yielding canvass with which the ceiling and walls were covered; the noises entering the windows when open for ventilation; the floorcloth with which for durability the House was covered, and the locking and opening of the doors. By lowering the ceiling (in order to get room for a foul-air chamber above,) less amount of voice became necessary to fill the house; at first from the same cause, the noise of conversation was louder. But the members soon learnt to pitch their voices in a lower tone, unless when talking for the express purpose of interruption. The walls were covered with reflecting wood panels, and the glass ceiling greatly assisted the reflection of sound; whilst the opening in the ceiling and the porous floor, allowed the useless impulses of sound to escape, and thus prevented offensive reverberations; for it is essential to the free sound of one syllable, that the remaining sound of the preceding one should be discharged, and not interfere with it. The unity of the temperature promotes unity of sound; the haircloth carpet prevents noise, and the double windows which have not been opened for seven years, exclude it.

Dr. Reid has had personal experience in practical acoustics. As a teacher of practical chemistry in Edinburgh—and a more laborious, earnest, and indefatigable one never lectured even in that industrious school—he had to address successive classes for seven and eight hours daily; and he built his classroom so as to enable him to do this with the least labour. He depended on a low and reflecting roof, the reduction of the walls to the least height compatible with the wants of the place, and on a rough absorbing earthen floor, and so successful was he, that whether his classroom were crowded or empty, either a whisper or the loudest sound was heard distinctly without any offensive reverberation, and he was enabled to lecture without raising his voice above the conversational tone.

The remainder of the volume is occupied with observations on the ventilation of theatres, coaches, ships, and mines, with plans and diagrams. The remarks on the ventilation of ships seem well worthy the attention both of the medical officers of the navy, and of that numerous body who are now engaged in the capacity of surgeons to merchant vessels and steam-ships. The proper ventilation of mines is of vast importance from the numbers who are engaged in them, and from the impure air to which they are exposed. Indeed it is a question, says Dr. Reid, whether more lives are not lost from impure air undermining the constitution gradually than from explosions and other accidents. The benefit of the Davy lamp in saving lives cannot be denied, but its benefits are lessened if it is used not merely as a protection from momentary dangers, but to enable the miner to work in a less wholesome atmosphere, by affording him light in air highly charged with a deleterious gas.

Dr. Reid has devoted a chapter to the consideration of the mutual relations of architecture and ventilation. He is justly of opinion that ventilation will never be placed on the footing which the progress of science, and the claims of health equally demand, till the means for ensuring its efficient application, shall be incorporated with the original design in all architectural structures, whether habitations, public buildings, ships, or manufactories. He adverts in strong terms also to the great importance of introducing among all classes of society a more precise knowledge both as to the movements of air, and the quantity necessary for respiration, urging the general introduction of select lessons in practical science in all schools and academies, as the only certain means of promoting equally the introduction of the proper measures by architects and their subsequent management by those who are more immediately affected by them. The book as a whole is rather deficient in method and unity, which could only have been attained by more time in polishing, condensing, and rewriting than its author probably had to bestow. Its matter, however, is good. It is written not to gain experience, but to give the result of experience, and we cordially recommend it to all who are interested in the subject, The reading of medical men is from their occupations so limited to what seems of immediate practical interest, that comparatively few would go through a thick octavo volume on ventilation. To these who would be deterred, we have supplied an analysis of those parts which they would have been interested in from their immediate practical bearing, had they been the readers instead of ourselves. If the result is merely to lead them to open their own windows more frequently, to be more observant of the air of their patient's rooms, to remove those mischievous chimney-boards from the fireplaces of their sick patients' bedrooms, and to supply the Toynbee ventilators* to the windows of the poor, who are confined in small rooms as more essential than any medicines, it will not have proved a useless one.

---

### Art. II.

* See British and Foreign Medical Review for October 1844, p. 501.

